# The Young Stethoscopist, or the Student’s Aid to Auscultation

**Published:** 1846-09

**Authors:** 


ART. V.—*The young Stethoscopist, or the Student's aid to Auscultation, by* HENRY J. BOWDITCH, M. D. *Boston, Wm. D. Ticknor & Co.* 1846, 12*mo.*, *p.p.* 278.

We are disappointed in this little volume: not that it has less value and merit than could reasonably be expected from a work of its character and size, but we are disappointed that the author did not undertake a production of somewhat larger scope, embracing rational, as well as physical methods of diagnosis. Of the ability of Dr. Bowditch as a practical Auscultator we had already entertained a high estimate. We were aware that to this branch of our art he had for many years devoted particular attention, and had enjoyed all the advantages of Parisian instruction and illustrations, as well as large opportunities for practical familiarity with the subject at home. We knew that his opinion was deemed high authority in pulmonary and heart diseases by his professional brethren of Boston, where those distinguished for their familiarity and skill with the stethoscope are not few and far between. We had consequently felt great desire to see the book so soon as we had learned its announcement; and we are, as we have said, disappointed to find it limited almost exclusively to physical exploration, and the facts, for the most part, compressed so succinctly in short, dry rules. We can readily conceive that studied in connexion with his oral teachings, the deficiencies of which we complain, would not be felt; but for the benefit of those of us who have not this advantage, we feel sorry that he did not allow himself more space and freedom — in fact, that he did not give us more of what we know enters into his oral instructions. We regret this the more, because our confidence in the ability of Dr. B. to write just the book we had hoped for, is not impaired, but enhanced by an examination of that which he has written. Our dissatisfaction, however, may not be in very good taste; for it may be said with great propriety that it becomes us to be sufficiently grateful for what we have, instead of finding fault because we desire more. As it is, we deem the work a valuable and useful production, for we feel assured that the instructions, so far as they go, are based on the author's own experience and judgment; and the satisfaction with which we have read the few passages in which he allows himself more latitude than usual, makes the general conciseness doubly aggravating.

With this captious preliminary, we proceed to avail ourselves of some extracts which we have marked as possessing especial interest and importance.

The following quotations from the preface we are happy to adduce, as testimony from a high source, respecting the proneness of some hyper-stethoscopists to mystify and confuse the subject by needless refinements; and, also, respecting the necessity of studying physical and rational diagnosis conjunctively:

"But I have had another, and perhaps equally important end in view, viz: to make you feel that the *time*, the *place*, the *circumstances* in which you may meet with these morbid signs, and the relations which they bear to the rational signs, are of as much importance as the physical signs themselves. There have been, I think, too many minute distinctions in regard to the particular sounds, a diffuseness of detail that has served to discourage rather than to allure you onward in the study of the interesting art of auscultation.

"In conclusion, while presenting this little work on the physical signs, let me disclaim all intention of placing them higher then they really deserve. Fifteen years ago, they were sneered at by many persons. Now, very few would be foolish enough to do so, and the tendency is strong to overrate them. Amidst the niceties of our physical examinations we are apt to neglect the rational signs. The truth is, that he who scoffs at either must necessarily be a child in the diagnosis of not a few diseases; and he who cultivates both with the clear, keen-sighted eye of a true observer, and then notes their mutual relations, is the truly wise physician. Both categories of signs are useful, each in its own sphere; and neither should be allowed to predominate; neither should be neglected."

We find in the progress of the work several passages which evince the desire of the author to simplify as much as possible, by repudiating distinctions which are more nice than wise. Our readers are aware of the views which we entertain on this point. (See Buff. Med. Jour. for Aug.)

We quote the following:

"Finally: it is of no importance for the pupil to trouble himself to decide, definitely, whether he hears *bronchophony*, *hægophony*, or the various kinds of *pectoriloquy*. It is sufficient that, on a comparison between the lungs, he finds an *increased* or *diminished* natural resonance in any part. The other physical and rational symptoms, when combined with even these, apparently, doubtful signs, will enable him to arrive at a correct diagnosis. He must, however, bear in mind the different sizes and shapes of the two primary bronchi; which facts usually cause *a little more vocal resonance at the top of the right lung*, than at the top of the left lung."

"Notwithstanding the marked difference between the *crepitous rale* and *gurgling*, the intermediate degrees are often difficult to be distinguished; and *these differences are of no real importance*, because they will be connected with other signs, which will aid the diagnosis.

"REMEMBER THIS RULE. *Do not trouble yourself so much about nice distinctions of sounds; but observe accurately*, first, *where the sounds are heard*; second, *where the focus of them is, supposing that they exist everywhere in both lungs; and* third, *their combinations with other physical and rational signs.*"

On the subject of percussion the following observations deserve to be remembered. Speaking of percussing the *tops of the shoulders*, he says:

"They are most unwisely neglected by some ascultators, as giving no definite results. In all cases of chronic cough I regard them as the most important, for they are the first affected in the earliest stages of phthisis."

Again, respecting minute shades of difference between the two sides of the chest, which, in certain cases, it is of great consequence to determine:

"A *difference of note* between two corresponding parts is not uncommon, when there is no real flatness in either. It occurs in cases in which the lung is not by any means impervious to air. Sometimes in the early stages of phthisis or pneumonia in its early or latest stages."

The following is perhaps the most valuable portion of the work. Every practitioner is aware of the great difficulty of making a diagnosis in many cases of suspected tuberculosis of the lungs. How often do we have the inquiry put to us with the most intense earnestness by patients and friends, "is the disease consumption." It is in such cases often, that positive auscultatory signs are most defective. Evidence from all sources is to be carefully collected and nicely balanced, before coming to a decision which is of such immense moment to those awaiting our opinions. Difficult and embarrassing as the question frequently is, we venture the assertion that a sufficiently comprehensive and minute investigation of all the circumstances directly or indirectly bearing on the case, will rarely fail to authorise a positive conclusion, without waiting to be guided by the effect of remedies, or the farther progress of the disease until, while all uncertainty is at length removed as to its character, the condition of the patient is utterly hopeless. We commend the following to the careful attention of the student and practitioner, as presenting the general method of investigation prosecuted by one of the masters of diagnosis :

"162. Example of chronic phthisis. As it is impossible to give any definite period, at which the peculiar signs occur; I will briefly give you an idea of my method of examining any case of chronic cough, in which I *suspect* phthisis. In the first place, I spend from twenty minutes to half an hour in learning the exact condition of the patient; his previous health from early age; his hereditary tendencies; the various diseases he has been subject to during his life; his profession and the influences of that upon his health. Having learned these and considered their bearing as predisposing causes of phthisis, I endeavor to fix the exact commencement of his actual disease, and to discover whether it showed itself, first by symptoms in the chest or elsewhere. After fixing those dates, I trace the different phases of the disease and general state of all the functions up to the moment of examination. Finally; I examine the cerebral, thoracic and abdominal functions in their actual condition. In doing all this, I may date back the commencement of the actual disease for weeks, months, or even years. I proceed then to the physical signs. In order that we may be more definite in our views, let us suppose that a man has had cough for some months; that he has or has not had hœmoptysis; that he has or has not had trouble in his digestive functions. Let us suppose that he has emaciated somewhat; has lost strength, but is still able to attend to his work. Let us imagine, moreover, that occasionally he has had slight "rheumatic pains," as they are called, about either of his sides or shoulders. In a word ; let a man be affected with *any* rational symptoms that could be referred to tubercular disease of the lungs. I then strip the chest wholly bare, and make the patient sit or stand very erect with the hands hanging by the side. If the clavicles are prominent ; if the intercostal spaces are contracted ; if the chest is flattened in front, I *fear* phthisis. If I see one clavicle much more prominent than the other, I fear still more that, in the apex corresponding to the most prominent clavicle, I shall find tubercles ; more especially, do I anticipate this result, if the "rheumatic pains," mentioned above, have generally been seated in that part. In regard to the movements, constrained or otherwise, of the parietes of the upper part of the chest, I pay but little attention though they may at times be of service. I observe whether one shoulder is higher than the other ; whether the ribs have fallen in behind, causing the scapula to fall towards the vertebra, and whether the nipple is depressed, all of which are the signs of old general pleurisy. Having inspected sufficiently, I proceed to percussion.— I examine, with especial care, the clavicles, in order to discover the slightest difference of sound between corresponding parts of each clavicle. Any degree of dullness, even the slightest *difference of note, if confined to the upper part* of the chest, between two portions equidistant from the spine or sternum augments my *suspicion* of the existence of tubercles. I care not whether that dullness be found on a part of the clavicle, or a spot only an inch square, at the top of the shoulder, over the scaleni ; if there be a decided difference, on repeated trials, my faith is, as mentioned. If there is a great dullness, for two or three inches from the top, while elsewhere percussion gives a clear sound, I am almost sure there is tubercular disease. If, on the contrary, the dullness extends all over one lung, I am doubtful if it be not the result of former pleurisy. If the dullness is at the base of the lungs, and the upper part is clear, I am morally certain that the disease is not tuberculous; i. e. if the patient is an adult. If it be a child, there is some doubt, because tuberculous disease sometimes attacks the lower part of the lungs in youth, while the upper portions are healthy. Having noted *thoroughly* all these results of percussion, even in their minutest shades, I proceed to the auscultation of the respiratory murmur. My object is to examine the inspiration and expiration, to observe whether their ratio is normal; whether one is shorter or longer than it ought to be; whether the inspiration is full and expansive, corresponding in duration to the movements of the chest, or whether it is shortened, so that the parietes are felt to rise and to expand considerably under the ear before the vesicular sound is heard; whether there be any prolongation of the expiration; whether either of these acts are jerking, as from some obstruction to the passage of air; whether there is the slightest rale, of any kind, with either, and, if there be, whether it is limited to the upper parts of the chest. If either of these conditions occur, and the results of auscultation and percussion agree, even in the slightest degree, my *suspicions* are *confirmed.* For example; let us take the most simple case. Suppose I find a difference of note; the right lung, at its apex, resounding less well than the left; if, in the same spot, I find the inspiration shortened, in a case where the rational signs point to phthisis, I feel certain of the existence of tubercles. If, however, the rational signs do not indicate the existence of phthisis, I shall have doubts about the diagnosis; but I shall have, nevertheless, very great fears that there are a few tubercles near the part. But, suppose I find absolute dullness and evident crackling; then I am sure of tubercles being there in a softened state; the forerunners of a cavity with gurgling and pectoriloquy.

In these examinations, I auscult during the common measured breathing which the patient is accustomed to. Having thus examined the whole chest, I try the effect of long breaths, at stated intervals, upon all parts that I have previously examined. Thus, I

frequently am able to find some of the differences in the murmur, or some of the rales, mentioned above, when nothing marked can be discovered in common breathing. In making the patient do this I am careful that the sound from the mouth, simulating bronchial respiration, is not transmitted. Many gross errors arise from neglect on this point. Having tried long inspirations, I make the patient cough and immediately inhale deeply, and listen to that and the rales which it sometimes produces. Through these remarks I have supposed I am examinining a doubtful early case of phthisis. But the rational signs may be very manifest; hœmoptysis, hectic fever, emaciation, &c. being present. I may then obtain greater differences on percussion; or if there is not any manifest difference on percussion, owing to the equal development of the disease in both lungs, I may still decide unfavorably for my patient, for, very likely, the respiration may have become *rude*, or coarse, which indicates there under the ear, much more of tubercular disease than of healthy lung. In others I may find that the lung is greatly solidified, causing an absence or great diminution of the respiratory murmur; or, possibly, a distinct bronchial or tubal sound may be heard, indicating a small cavity, with possibly condensation about it. Finally, I may find a cavernous sound, which becomes to me an indication of a large excavation with thin parietes. Connected with these variations in the murmur, are the rales I frequently hear. For example, a slight sibilant or sonorous rale, or a single click is often heard in the *rude* respiration; and it is then indicative of condensation without excavation; a crackling with the simple tubal, indicating softened tubercles, and finally gurgling with the tracheal or cavernous, indicating large ulceration. I remember that frequently these rales are produced only by a full breath, or a cough. Hence I cause the patient to cough, and generally perceive the importance of the act, as I have already stated elsewhere. In addition to the production of rales the cough is useful in itself as a means of testing the resonance; I therefore notice the amount of that *resonance* in the same manner as I notice the resonance of the voice.

If, in *any* phthisical case, I find marked resonance of the voice, or any manifest difference between the two apices, except, perhaps, a little more resonance at the top of the right than of the left, my prognosis is very unfavorable, for a very general condensation by tubercles is indicated, or what is more common, a cavity, either larger or smaller, in the portion of the lung under the ear."

We pass to *Pericarditis*, a disease which is by no means so rare as would appear from the infrequency of its diagnosis. The attention of the profession has of late years been called to the frequency of its occurrence as a complication of acute articular Rheumatism. But it may be developed irrespective of this connection, and is very liable to be mistaken. A case may deceive, for example, where the prominent symptoms are pain, but not so severe as in pleuritis, and great disturbance of the action of the heart; but the pain not being distinctly aggravated by respiration, the disease is diagnosticated and treated as a neuropathic disorder. In the mean time incurable lesions are produced,and the nature and seat of the disease are revealed by an autopsy. We have known precisely such a case. We are fearful that it is not injustice to the profession to say that a large proportion of cases of this disease are only detected until it is too late for active treatment. This is the more lamentable, because there are few forms of disease where prompt and efficient treatment is of greater importance to the safety of the patient. The following embodies a summary of the circumstances chiefly concerned in the diagnosis:

"Nothing is more indefinite than the *rational* signs of pericarditis. It is impossible to be sure of its existence, unless we have recourse to physical signs. A most common case is as follows. A man is seized, either when healthy, or, more commonly, when affected with rheumatism, with a pain, either very severe or very slight, about the cardiac region. This is the more common case. At times, however, there is no pain. Dyspnœa may come on, and, though it is usually slight, it may become orthopnœa. With these *may* come other, more or less marked, signs of trouble in the circulation; such as dizziness, œdema of the legs, &c.; but all these symptoms *may* be absent, and the disease would be *wholly latent* if we did not have the physical signs to inform us of its existence. If you happen to see the patient quite early, you may obtain only signs of some obscure disease about the circulatory system; but of what character you may be doubtful. Any alteration either of the sounds or of the impulse, any irregularity of the rhythm, &c. may lead you to *suspect* pericarditis or endo-carditis. If *any* physical sign be combined with any local rational sign, such as a pain in the region of the heart, or palpitation, &c., your suspicions would be augmented. If, in connection with these signs, or without them, you hear a superficial *creak* like the sound of *new leather*, you may be certain of your diagnosis, and decide that it is a case of pericarditis. Yet it is quite rare to hear this sign; and if it be absent you must not necessarily infer that pericarditis does not exist. If you hear it, you will, in your mind's eye, see the two layers of the pericardial sack, congested and drier than usual, and hence moving with less ease than ordinary; yet not sufficiently obstructed to prevent the next phenomena, viz.: *rubbing sound*, which may come on within forty-eight hours from the first attack. Hearing this, either locally, about the third and fourth rib, near the sternum, where it would be most likely to commence, or, generally, over all the cardiac space, you may be morally certain that you have pericarditis to deal with, and that the opposing layers of the pericardium are even then covered with a tolerably dense, and moist membrane, the motions of which, upon each other, produce the sound in question. This is the last moment for active, efficient treatment, if you have not used it before. At this period you may perceive some obscurity in the movements of the heart; little or no increased dullness on percussion. In a few hours, if the disease be not checked, an effusion of fluid will take place and will separate the layers, push back the heart, destroy the rubbing sound, and you will have instead, great diminution in the strength of the sounds of the heart; absence of impulse; perhaps a sort of churning or washing sound, in consequence of the dashing of the fluid against the walls of the distended sack; dullness on percussion, which may extend from the second rib to the cartilages, and from the junction of the cartilage and right ribs to beyond the nipple of the left side; and, finally, an absence of respiration over a large part of the left breast (owing to the pushing aside of the lung,) with prominence of the same point. These are the most perfect and undoubted physical signs of pericarditis. If the disease is prepared to yield, all these signs will diminish, the prominence will disappear, the dullness on percussion will diminish; perhaps the rubbing sound will be again heard, and then it will be very favorable, as it will indicate an absorption of the fluid. It will usually commence near the origins of the aorta and pulmonary artery, and will extend thence over the cardiac space, and may last for weeks; the impulse and the sounds of the heart will be felt and heard more distinctly, and gradually all symptoms will disappear. But this is not the common course, for palpitation usually remains for some time, and not unfrequently will this symptom go on augmenting until the patient will finally sink under the signs of confirmed hypertrophy of the heart.

"*Chronic Pericarditis* presents very much the same signs as those described for the acute disease, therefore it is useless to speak of them. Moreover, they are frequently connected with hypertrophy."

The remarks preliminary to the foregoing extract will apply measurably to Endo-carditis. The former, in fact, co-exists to a greater or less degree in the majority of cases of the latter disease. We quote the author's observations:

"This disease is most frequently the result of a rheumatic inflammation of the lining membrane of the heart. Not uncommonly, during this disease, the pains will leave the extremities, and a dull aching sensation, and, more rarely, a severe pain, will be felt in or near the cardiac region. It may be so slight, that, frequently, the patient will neglect to speak of it, and will think himself wholly recovered, so freely is he able to move. Thus daily auscultation is necessary; and, if endo-carditis is commencing, you will probably hear some prolongation of the first sound. This may, perhaps, be checked and your patient may get well; the bellows murmur may either subside entirely, or may remain, as a mere trifle, not sufficient to impair the health. This period you may overlook, or you may be unable to check it, and your patient, in a few weeks, will probably complain of palpitation and of some dyspnœa. The bellows murmur then will have become more rough and the impulse stronger. Remedies may relieve, but they rarely cure in this stage of the disease; for the lymph deposited on the valves, &c., will have too firmly obstructed the valvular apparatus. The disease may continue for months, and the patient may have the same physical signs and no great deterioration of health; or he may, from inattention to the rules of health, become more ill, and be finally affected with hypertrophy of the heart and the symptoms usually following in the train of this combination. This may be given as an example of an acute attack, but the majority of cases progress more quietly and the signs are less distinct.

The following painfully interesting illustration of the importance of employing auscultation in determining the fact of pregnancy in certain medico-legal cases, we take from the appendix. For this, if for no other application, obstetric ausculation possesses imperative claims on the attention of every member of the profession. Who can think of the possibility of being, positively or negatively, accessary to a repetition of a similar act of legal barbarity without a shudder!

Among the most deplorable cases on record, in which innocent human life was deliberately taken, according to legal forms and by the adjudication of the supreme tribunals of the land, that of the child of Mrs. Spooner, who was executed July 2, 1778, stands preeminent. Under our modern means of diagnosis of the earlier periods of pregnancy, especially with our knowledge of obstetric auscultation, such a case I presume will never happen again. It impressed me most deeply, for many reasons; but I quote it now because it may stimulate you to the study of the minute stethoscopic phenomena of early pregnancy. You may at any time be placed in circumstances in which the knowledge of these may be of the highest importance to you. Mrs. S. was convicted of having been an accomplice in the murder of her husband, and after having been condemned she begged a *reprieve*, on the ground of being "several months advanced in pregnancy." The execution was stayed one month, and the court ordered a jury of "matrons," or, in other words, of ignorant old women to be summoned to decide the question, whether or not she was "quick with child." This practice, a relic of antiquated absurdity, was put in execution, and these twelve

*matrons wise in medical diagnosis!* with two menmidwives, decided that she was *not* "quick with child."* Mrs. Spooner immediately sent to the council a most touching petition in which she says, "although the jury of matrons have not decided in my favor, still I am absolutely certain of being in a pregnant state, and above *four months* advanced in it, and the infant I bear was lawfully begotten. I am earnestly desirous of being spared till I am delivered of it. I most humbly desire your honors, notwithstading my great un[illegible]orthiness, to take my deplorable case into your compassionate consideration. What I bear and clearly perceive to be animated, is innocent of the faults of herwho bears it, and hath, I beg leave to say, a right to the existence God has begun to give it. Your honors' humane, *Christian* principles, I am very certain must lead you to desire to preserve life, even in this, its miniature state, rather than to destroy it. Suffer me, therefore, with all earnestness, to beseech your honors to grant me such a further length of time, at least, as that there may be the fairest and fullest opportunity to have the matter fully ascertained, and, as in duty bound, shall during my short continuance, pray." Notwithstanding this urgent appeal of a mother in behalf of her innocent unborn babe, the honorable council of Massachusetts ordered her execution, and thus inflicted a stain that can never be eraced from the annals of the jurisprudence of the State; and they did so, notwithstanding that two men midwives and one of the "*matrons*" certified that they believed that Mrs. S. was quick with child, and that they had been mistaken in the verdict they had previously given! It was sufficient that "Elizabeth Rice and Molly Tattman" certified to the contrary, and that they believed her to be "not even now (June 27, 1778) quick with child."

With indecent haste, amid the fury of the elements, and the shouts of the crowd, this legal murder was consummated. On the evening of the day of the execution, the "body was examined, as the prisoner had requested, and a perfect male fœtus of the growth of five months was taken from her!"

As I have already stated, I quote this case chiefly to show the importance of every sign of early pregnancy in medico-legal questions. I have no hesitation in believing that, had auscultation been known and practiced on that occasion, it would have revealed the existence of the sounds of a fœtal heart, and this sad event would have been prevented. Does not the reverse of the fact prove to the medical student the importance of accurate obstetric auscultation? For though we have improved somewhat in the forms of jurisprudence during the last seventy years, we are still liable to have the folly of a *matron* jury enacted over again by the advice of the wisest on the bench. The student of medicine ought always to protest against such an absurdity, and to claim for the auscultatory

*American Criminal Trials, by Peleg W. Chandler, Boston: T. H. [illegible]arter & [illegible]o. vo.ii. 1845.

signs their relative and important place among the phenomena of early pregnancy.

In conclusion, we would say to the *Young Stethoscopist*, whether pupil or practitioner, that he will do well to purchase Dr. Bowdich's book. The rules and suggestions are to be received with confidence, as the results of extensive and reliable practical experience. We complain only of their brevity, and the exclusion of symptoms, which we think should never be dissevered from the signs. We trust that in the second edition, which we presume will soon be called for, the author's modesty or prudence will permit him to expand the scope of the work so as to do the subject and himself better justice.

